# Effectiveness of outpatient parenteral antimicrobial therapy (OPAT) for patients with cranial infection

**DOI:** 10.3389/fmed.2023.1202969

**Published:** 2023-10-24

**Authors:** Fatma Kilinc, Matthias Setzer, Bedjan Behmanesh, Daniel Jussen, Florian Gessler, Simon Bayerl, Volkhard A. J. Kempf, Johanna Kessel, Marcus Czabanka, Vincent Prinz

**Affiliations:** ^1^Department of Neurosurgery, Goethe University Hospital, Frankfurt am Main, Germany; ^2^Department of Neurosurgery, University Medicine of Rostock, Rostock, Germany; ^3^Department of Neurosurgery, Charité-Universitätsmedizin, Berlin, Germany; ^4^Institute for Medical Microbiology and Infection Control, University Hospital Frankfurt, Frankfurt am Main, Germany; ^5^University Center of Infectious Diseases, University Hospital Frankfurt, Frankfurt am Main, Germany; ^6^University Center of Competence for Infection Control of the State of Hesse, Frankfurt am Main, Germany; ^7^Department of Medicine, Infectious Diseases Unit, Goethe University Hospital, Frankfurt am Main, Germany

**Keywords:** cranial infection, intravenous antimicrobial therapy, hospitalization, OPAT, efficacy

## Abstract

**Objective:**

Outpatient parenteral antimicrobial therapy (OPAT) is a well-established and cost-effective method for improving the efficient use of healthcare resources. However, only a few centres in Germany perform it. Here we analysed OPAT for the treatment of patients with cranial infections in our neurosurgical department.

**Methods:**

This retrospective study analysed patients with cranial infections and the need for intravenous (i.v.) antimicrobial treatment between 2018 and 2021.

All diagnosed intracranial infections were defined into two infection categories such as long-term antimicrobial treatment and short-term antimicrobial treatment. All included patients were discharged with a peripherally inserted central catheter (PICC) line. Prior to discharge, all patients received training in the safe administration of their medications *via* the PICC line. The duration of OPAT and the rate of readmission after OPAT were analysed.

**Results:**

We identified a total of 45 patients treated with OPAT for cranial infections. Intradural involvement was present in 40 cases (88.9%). The average length of hospital stay for this cohort after surgical treatment was 45 ± 15 days. 5 patients were treated for soft tissue/skin infection. Surgery was not required in this cohort. The mean hospital stay for this cohort was 8 ± 6 days. Gram-positive organisms were isolated in most cases (53.3%). The most common pathogens were *Staphylococcus aureus* followed by other *Staphylococcus species*. For all included patients, OPAT was performed after discharge for an average of 43.1 ± 14 days. There were five cases of readmission due to treatment failure. No serious adverse events or complications of OPAT were observed.

**Conclusion:**

OPAT enables better patient-centred healthcare close to home. The length of hospital stay can be reduced and adverse events due to prolonged hospitalisation can be avoided.

## Introduction

Cranial infections are server and potentially life-threatening conditions that demand prolonged intravenous antimicrobial therapy and extended hospitalization. However, for some patients there is an alternative approach known as outpatient parenteral antibiotic therapy (OPAT) which enables them to receive intravenous antibiotic treatment at home without the need of hospitalisation (1). This is particulary relevant for complex cranial infections, such as brain abscesses or cases following brain surgery, such as tumor resection or decompression in traumatic brain injury, where 4–6 weeks of parenteral antibiotic treatment may be required, depending on intra- or extradural involvement (2, 3).

Numerous studies have already established OPAT as a safe and effective treatment program providing patients with the opportunity to complete their treatment safely and effectively in the comfort of their own homes (4–8). The benefits of OPAT extend to both the patients and the healthcare providers, as it reduces length of hospital stay, enhances patient satisfaction and decreases overall treatment costs (6–8). Although OPAT is well established and widely used in the healthcare system in countries such as the UK and the USA, it is still performed in only a few centres in Europe. Comprehensive data regarding its prevalence and effectiveness are still lacikng (8–12).

To the best of our knowledge, there are no published data specifically focusing on the use of outpatient parenteral antimicrobial therapy for cranial infections. Therefore our aim in this study was to analyse the safety, feasibility and efficacy of OPAT in patients with cranial infections, addressing this gap in knowledge.

## Methods

### Study design and population

In this retrospective study, patients with cranial infections requiring intravenous (i.v.) antimicrobial treatment via OPAT between 2018 and 2021 were analysed by reviewing medical reports and outpatient records. All patients were treated at the neurosurgical department of Goethe-University Frankfurt.

### Inclusion and exclusion criteria

**Figure 1:**
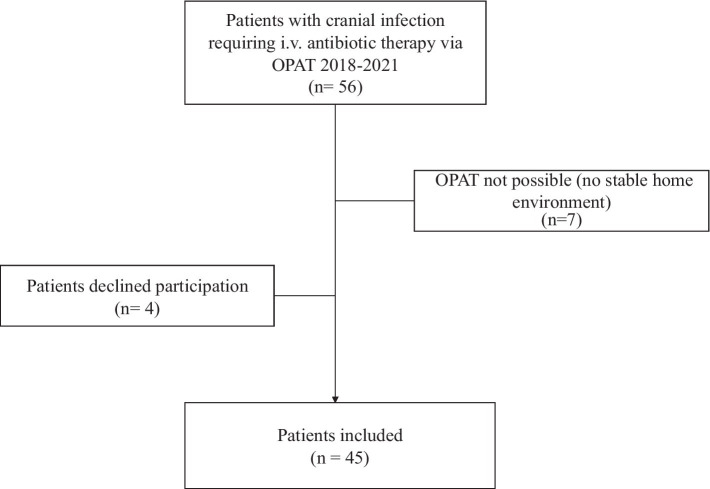
Flowchart describing the inclusion process of patients with cranial infection.

All patients with a cranial infection aged over 18 years were included. Patients who could not be discharged home with OPAT due to lack of a safe social environment were excluded, as were patients who were unable to self-administer intravenous antibiotics (Figure 1).

Swabs were taken from each patient intraoperatively to determine the presence of bacteria (13, 14). Blood cultures were also taken from each patient before intravenous antibiotic therapy was started. All laboratory testing was performed under strict quality-controlled DIN ISO 15189:2007 standards (certificate number D–ML–13102–01–00) as formerly described (15). For MRSA screening, nasopharyngeal swabs were routinely obtained. Screening for multidrug-resistant gram negativ bacteria (e.g., ESBL) and VRE was done by collecting rectal culture swabs with Amies collection and transport medium. If applicable, swabs from wounds or tracheal secretion were also taken.

Antibiotic-resistant organisms were defined as methicillin-resistant *S. aureus* (MRSA), vancomycin-resistant *Enterococcus faecium* (VRE), and extended-spectrum beta-lactamase-producing *Escherichia coli* and *Klebsiella pneumoniae* (ESBL) (13, 14).

### Data collection and analysis

Patient records were analysed for baseline data such as age, sex, infectious disease diagnosis and treatment characteristics, specifically antimicrobial name, class and duration. Outcomes such as readmission, return to the emergency department and patient satisfaction were also analysed. All diagnosed and analysed cranial infections were defined into two infection categories according to long-term antimicrobial treatment (intradural) or short-term antimicrobial treatment (extradural) involvement. When bacteria were identified, broad-spectrum/calculated antibiotic therapy was changed to targeted culture-specific sensitive antibiotic therapy. Reports of laboratory values and changes in orders were made by telephone, e-mail or in person.

Patients were enrolled in the OPAT service if they lived in a stable home environment, were able to understand the implications of the program, and had easy access to medical care when needed. Self-administered OPAT was used for all patients enrolled and analysed, with the healthcare professional assisting the patient and/or family members with the antibiotic regimen. Prior to discharge, all patients received a peripherally inserted central catheter (PICC) line and training in the safe administration of their medication via the PICC line. In addition, before discharge, patients were trained to apply antibiotics, start the infusion process and remove the PICC line. It was also checked before discharge whether the patients were in a safe social environment. The physical presence of healthcare staff was not required during antibiotic administration. To detect complications and for assessing safety of OPAT, patients were seen at least once a week after discharge for clinical and laboratory checks. Patient satisfaction after OPAT was assessed by a standardised interview at the final follow-up.

## Results

We identified a total of 45 patients with a median age of 49 years (range 20 to 71 years) who were treated for a cranial infection *via* OPAT between 2017 and 2022. The gender distribution for the entire cohort showed a male predominance, with 60% being male (Tables 1, 2).

**Table 1:** Demographic data and treatment.

Sex		*n* = 45
Female		18 (40%)
Male		27 (60%)
Mean age, yrs		49
Operatively treated patients		
Sex		*n* = 40
Female		17 (42.5%)
Male		23 (59%)
Mean age, yrs		49
Duration of treatment after discharging (mean, days)		45 ± 15
Diagnosis		*n* = 45
Brain tumor		
High-grade		20 (44.4)
Low-grade		7 (15.6)
Epidermoid		2 (4.4)
Metastasis		3 (6.7)
SAB		4 (8.9)
Shunt		3 (6.7)
Pituaitary surgery		3 (6.7)
Cavernoma		3 (6.7)
OPAT antibiotics (number of episodes (%))		
Cefazolin		5 (12.8)
Fosfomycin		12 (30.8)
Meropenem		12 (30.8)
Vancomycin		10 (25.6)
Penicillin		2 (5.1)
Ceftriaxone		9 (23.1)
Ceftazidin		2 (5.1)
Gentamycin		1 (2.6)
Cefotaxim		2 (5.1)
Clindamycin		3 (7.7)
Clindamycin		3 (7.7)
Conservatively treated patients		
Sex		*n* = 5
female		1 (20)
male		4 (80)
Mean age, yrs		40
Extradural infection		
High grade brain tumor		3 (6.7)
Low grade brain tumor		1 (2.2)
Cavernoma		1(2.2)
OPAT antibiotics (number of episodes (%))		
Cefazolin		2 (40)
Fosfomycin		1 (20)
Meropenem		1(20)
Ceftriaxone		2(40)
Duration of treatment after discharging (days)		8 ± 6

**Table 2:** Hospital stay and duration of treatement.

Operative	*n* = 45
Hospital stay	11.6 ± 10
Duration of treatment after discharging (mean, days)	45 ± 15
Conservative	*n* = 5
Hospital stay	8 ± 6
Duration of treatment after discharging (days)	8 ± 6

All diagnosed cranial infections were defined in two infection categories, such as intradural long-term antimicrobial treatment (intradural) or short-term antimicrobial treatment (extradural) involvement. Their demographic and clinical characteristics are shown in Table 1.

In 34 cases (86.7%), intradural involvement with subsequent bone graft removal was detected. The mean hospital stay for this cohort was 11.6 ± 10 days. Extradural involvement was seen in five patients. In these five patients, surgery was not necessary and conservative therapy with i.v. antibiotics alone was sufficient. The mean hospital stay for this cohort was 8 ± 6 days. In the remaining 3 patients, intradural infection was detected after shunt implantation. In all 3 cases the shunt was removed. In three other cases, infection was detected after pituitary surgery. In these cases, only conservative i.v. antibiotic therapy was required without a second operation.

The majority of patients (53.8%) were treated with two or more drugs after surgery for the duration of the OPAT. The most commonly prescribed antimicrobials were meropenem (30.8%) and fosfomycin (30.8%), followed by vancomycin (25.6%).

In most cases of conservatively treated patients one drug for the duration of OPAT was used. The most commonly prescribed antimicrobials were cefazolin (40%) and ceftriaxone (40%), followed by fosfomycin (20%) and meropenem (20%).

Gram-positive organisms were isolated in most cases (64.4%). The most common pathogens were *S. aureus* followed by other *staphylococci.* Multi-resistant gram-negative bacteria (MRGN) were detected in two cases. Antibiotic treatment was administered according to the results of the antimicrobial susceptibility testing (Table 3).

**Table 3:** Etiological pathogens isolated from blood cultures or intraoperative specimens.

Organism isolated	*n* (%)
*S. aureus*	24 (53.3)
Methicillin-sensitive *S aureus*	23 (95.8)
Methicillin-resistant *S aureus*	1 (4.2)
Other *Staphylococci species*	6 (13.3)
*Streptococci*	7 (15.6)
Culture negative	8 (17.8)

For all included patients, OPAT was performed for a mean of 43.1 ± 14 days after discharging. The duration of antimicrobial treatment for intradural involvement was 45 ± 15 days and for extradural involvement 8 ± 6 days.

### Outcome

Patients with intradural involvement required readmission due to treatment failure in 3 cases. Surgery was required in all 3 cases. After surgery, antibiotic therapy was escalated until the presence of bacteria from intraoperative swabs was detected. In two of these three cases, initial antibiotic therapy was with fosfomycin and cefazolin. One case was treated with penicillin. In these three cases, an additional bacterium was detected after the second operation, which had not been detected during the first surgery (Table 4).

**Table 4:** Readmission after discharging.

	*n* (%)
*Surgical treated patients*	
Readmission	3 (7.5)
Treatment failure	3
Surgery after readmission	3
Reason for readmission	
New detected bacteria	3
*Conservative treated patients*	
Readmission	2 (40)
Treatment failure	0
Reason for readmission	
Urinary tract infection	2

In patients with extradural involvement (e.g., soft,- and skin infection), two patients had to be readmitted. Relevant changes in laboratory parameters (increased inflammantory parameters) were noted. Surgical treatment was not required. The wound conditions were unremarkable. In both cases a urinary tract infection (UTI) was diagnosed as the reason for the increasing infection parameters. In both cases, the initial antibiotic treatment was cefazolin. After escalation of antibiotic therapy, the UTI was treated and the patients were discharged (Table 4).

To assess patient satisfaction after OPAT, standardised interviews were conducted at the last follow-up using a structured, one-time, detailed questionnaire. Questions were asked about general health, pain intensity, and general satisfaction in everyday and household life after OP. The answers to these questions were ‘yes’ or ‘no’. Patients were also asked about any problems with intravenous antimicrobial therapy and early discharge.

Seven patients were not able to be interviewed because they had passed away. The reason was an advanced high-grade brain tumor. Five patients were lost to follow-up. The mean follow-up was 18.7 ± 14 months. The majority of patients reported a high level of satisfaction, and the possibility of early discharge with return to family, home, and in some cases to work was described as positive (Table 5).

**Table 5:** Outcome after OPAT (*n* = 33).

Patients satisfaction during OPAT	28 (84.8%)
Readmission during OPAT	5 (15.2%)
Managing to do’s alone at home	19 (57.6%)

## Discussion

In our study, we analysed the safety and feasibility of OPAT, which has become the standard of care for patients with cranial infections in many countries around the world. There are several studies in the literature that support and describe the safety and efficacy of OPAT programs (1, 5, 11, 16).

The results of our study serve as an important reference for healthcare professionals and policy makers, providing reassurance and confidence in the effectiveness of OPAT as an alternative to traditional hospital treatment. This evidence can help shape clinical practice and encourage wider adoption of OPAT as a patient-friendly and cost-effective option, ensuring better health outcomes for patients with cranial infections. The data from our study are complemented by the study of Quintens et al. which also evaluated the efficacy and safety of OPAT in Belgium, further reinforcing the reliability of OPAT as an effective treatment option outside the conventional hospital setting (17).

The consistent findings from various studies, including those from Wai et al. and Chapman et al. consistently highlighted the patient-centered nature of OPAT, with treatment asministered at home. It also reduces the risks associated with long hospitalization and contributes to a high level of patient satisfaction. Moreover, early discharge facilitated by OPAT not only benefits patient well-being but also demonstrates cost-saving potential, making it an attractive option for healthcare provides (6, 10).

As known, parenteral antibiotics for cranial infections usually require prolonged intravenous treatment in hospital. While OPAT has ganined widespread recognition in countries lies UK or US, and despite the described clinical benefits of avoiding hospitalisation and keeping care closer to home, it is essential to address the absence of a uniform system of outpatient therapy centres for OPAT in Germany (5, 7, 8, 12).

For example, in the UK, OPAT was until recently limited to a small number of specialist centres. However, it has been expanding in recent years as its significant benefits to local health services and patients have been increasingly recognised. The duration of common infections treated with OPAT, such as bone and joint infections, skin and soft tissue, central nervous system, pulmonary, intra-abdominal and urogenital infections, ranges from 2 to 8 weeks or longer (6).

Chapman et al. describe OPAT as providing high quality, patient-centred, cost-effective and accessible care. In most cases and countries OPAT is used primarily for financial reasons (10).

Due to the DRG system and the different diseases and therapies of the patients, it was difficult to calculate the costs saved by early discharge.

Nevertheless, the additional cost of antibiotic therapy that could be saved by early discharge of patients with cranial infection was 1.255.607 Euro. Gonzales-Ramallo et al. also describe in their study that early discharge and intravenous therapy at home saved about 80% of the cost (18).

Appropriately, Durojaiye et al. also as well as Miron-Rubion et al. described the cost-effectiveness of early discharge and i.v.-home therapy (19, 20).

This study also presents some of the first published data on the use of OPAT for cranial infection in Germany. The results of the analysis clearly show that, despite the severity and complexity of cranial infections and irrespecitve of intradural or extradural involvement, it is possible to performe OPAT feasible and patient-friendly.

OPAT is all about providing high quality, patient-centered care at home while avoiding the risks associated with hospitalisation. These positive health outcomes should be used by OPAT clinicians/healthcare managers and policy makers alongside the already strong clinical effectiveness and patient safety data, to drive the further development of OPAT in Germany.

Our data cleary demonstrate that the OPAT service model used in our center is safe and clinically effective, with low rates of complications/readmissions and high levels of patient satisfaction.

In conclusion, this study shows that OPAT is safe for patients with cranial infections. The results of this study suggest that OPAT is a viable alternative to hospital treatment for a wide range of infections in appropriate patients. To the best of our knowledge, this is the first study to evaluate OPAT for the treatment of patients with cranial infections. It allows for better patient-centered healthcare close to home and can reduce the length of hospital stay and avoid adverse events due to prolonged hospitalisation.

### Limitations

This study is limited by its retrospective design and limited number of patients. A further limitation of this study is the fact that it is a single centre study.Most patients in our study had a long follow-up period. Our cohort may be subject to recall bias because of the time that elapsed between our evaluation and the time of OPAT. Possible complications or side effect of antibiotic therapy were not analysed.

## Data availability statement

The original contributions presented in the study are included in the article/supplementary material, further inquiries can be directed to the corresponding author.

## Ethics statement

This study was approved by the local ethics committee of the Goethe University Frankfurt am Main, the methods were carried out in accordance with the relevant guidelines and regulations. Informed consent, including the use of tissue samples, was obtained from all subjects and/or their legal guardian(s).

## Author contributions

VP and FK: conception and design, analysis and interpretation of data, statistical analysis. FK: acquisition of data and acquisition of data. VP, FK, and MC: reviewed submitted version of manuscript. VP: approved the final version of the manuscript on behalf of all authors. MS, BB, FG, DJ, SB, JK, and VK: administrative or technical or material support. MC: study supervision. All authors contributed to the article and approved the submitted version.
